# New Calibration System for Low-Cost Suspended Particulate Matter Sensors with Controlled Air Speed, Temperature and Humidity

**DOI:** 10.3390/s21175845

**Published:** 2021-08-30

**Authors:** Zenon Nieckarz, Jerzy A. Zoladz

**Affiliations:** 1 Faculty of Physics, Astronomy and Applied Computer Science, Jagiellonian University, ul. Łojasiewicza 11, 30-348 Kraków, Poland; 2 Faculty of Health Sciences, Jagiellonian University Medical College, ul. Michałowskiego 12, 31-126 Kraków, Poland; j.zoladz@uj.edu.pl

**Keywords:** calibration, low-cost sensors, air quality, particulate matter

## Abstract

This paper presents a calibration system for low-cost suspended particulate matter (PM) sensors, consisting of reference instruments, enclosed space in a metal pipe (volume 0.145 m^3^), a duct fan, a controller and automated control software. The described system is capable of generating stable and repeatable concentrations of suspended PM in the air duct. In this paper, as the final result, we presented the process and effects of calibration of two low-cost air pollution stations—university measuring stations (UMS)—developed and used in the scientific project known as Storm&DustNet, implemented at the Jagiellonian University in Kraków (Poland), for the concentration range of PM from a few up to 240 µg·m^–3^. Finally, we postulate that a device of this type should be available for every system composed of a large number of low-cost PM sensors.

## 1. Introduction

In the last years, several studies have shown evidence for a large potential impact of low-cost sensors as a tool for indoor and outdoor environmental studies concerning air pollution exposure and assessment of health risk in humans [1,2,3,4,5] and animals [6]. However, this type of sensor has demonstrated challenges that may include accuracy, reliability, repeatability and calibration [7,8,9,10]. For this reason, it is necessary to be able to initially and periodically verify readings of such sensors [11]. It should be noted that this issue becomes particularly important in the case of producing and using a measurement network where large numbers of such sensors are used [12,13,14].

While organizing and coordinating the work of the measurement network, one is almost certain to face the need to perform calibration (service, periodic or control) at different times of the year. Therefore, the use of a device and calibration procedure based on natural air could not constitute a sufficient procedure, since it is rather difficult in natural air to observe proper changes in PM concentrations in a short period of time, needed to perform a correct calibration procedure. For example, in the case of some cities where air quality can be strongly affected by the intensity and direction of wind [15,16], lack of pronounced changes over longer periods of time towards low and high levels of PM concentration in atmospheric air can disturb calibration procedures based on natural air.

In order to be able to perform calibration regardless of the quality of natural air and local weather conditions (wind, precipitation), it is necessary to have an appropriate laboratory calibration device. Ambient and laboratory evaluations of calibration systems for low-cost particulate matter sensors were performed previously [17,18,19]. Some of them are state-of-the-art laboratory chambers [20], which are accurate and precise, but extremely expensive. Others are calibration chambers, where temperature and relative humidity were controlled [14,21,22]. However, none of the known solutions provides testing of the PM sensors under known or controlled air flow velocity, with the only exception of the study by Spinelle et al. [23], who studied an NO_2_ micro-sensors in an “O”-shaped ring-tube system allowing, among others, air velocity be to controlled.

That’s why a proper calibration system should have the capacity of calibrating sensors at different known air flow rates. However, to the best of our knowledge, the available calibration systems do not have such an option. That’s why we also aimed in the described new calibration system at providing an option of sensor calibration at different air flows. Thus, our calibration system allows the user to perform sensor calibration at a chosen air flow. This seems to be a clear advantage of our system, when compared with other systems described in the literature.

It is worth noticing that meteorological conditions with air stagnation [24,25] or extremely low wind speed [26] are usually infrequent in real situations.

This paper addresses the issue of sensor calibration and presents in detail a simple and inexpensive system that allows one to study accuracy, repeatability and calibration of low-cost sensors of suspended particulate matter in a laboratory conditions with a given air flow velocity, known temperature, relative humidity and pressure. This system allows one to carry out a multi-point calibration process and obtain reliable results. Furthermore, this paper describes the mechanical structure of the device, sensors used and control method of calibration. Finally, this article presents the results of an exemplary calibration carried out for two university measuring stations (UMS) developed and used by the scientific project known as Storm&DustNet [4], implemented at the Jagiellonian University in Kraków (Poland).

## 2. Materials and Methods

**Figure 1:**
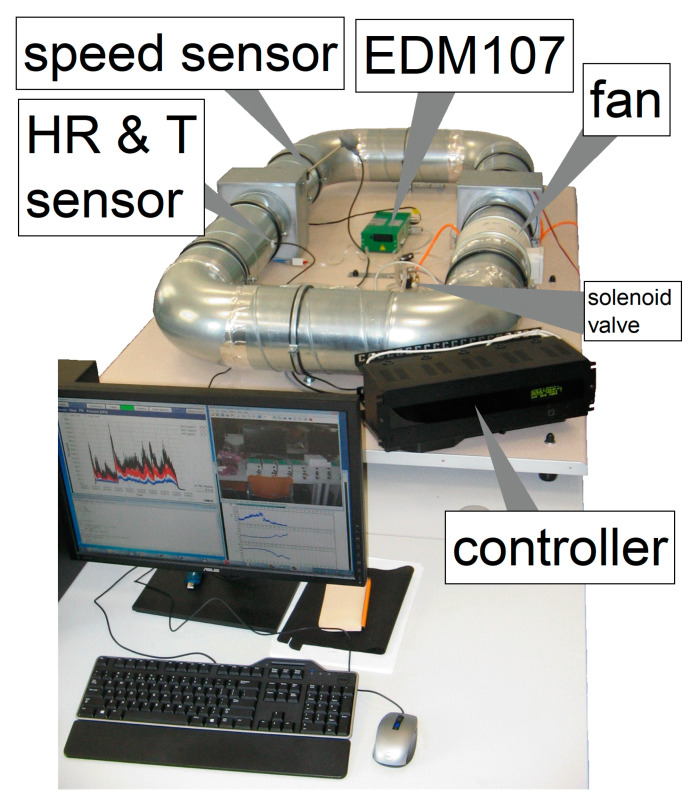
View of the calibration system.

The calibrator system was built as an enclosed space in a metal pipe (standard ventilation ducts) with a circular cross section. The device has a duct fan that provides air flow and circulation under control of a computer program. The diameter of the pipe and the fan equals to 200 mm and the total length is 6.5 m. The volume of the enclosed space equals to 0.145 m^3^. This length of the tunnel circumference and the air velocity of 0.65 m·s^−1^ gives a characteristic mixing time of 10 s. The calibrator system covers an area of 2.52 m^2^ (2.1 m × 1.2 m). An overview of the calibration system is shown in Figure 1.

The device has been equipped with air velocity transmitters (IVL10, PRODUAL), which are designed to measure air velocity and temperature inside the duct (±0.5 m·s^−1^ ± 7% accuracy of velocity from reading and ±0.5 °C accuracy of temperature), and an integrated temperature and humidity sensor (SHT75, SENSIRION) with the operating ranges: humidity from 0 to 100% (±1.8%); temperature: from −40 to +120 °C (±0.3 °C). The computer can: (a) read the data from the reference station with the measurement error amounting to ± 2 µg·m^−3^ (EDM107, GRIMM Aerosol Technik) and from both sensors; (b) controls the fan; (c) and the particulate matter injector.

It seems to be worth mentioning that it is possible in our system to configure wind speed at different levels, from 0.5 to 5.0 m·s^−1^, or even up to 7 m·s^−1^ (technically possible). The reference instrument collects particles of the size within the range of 0.25–10 µm in diameter.

**Figure 2:**
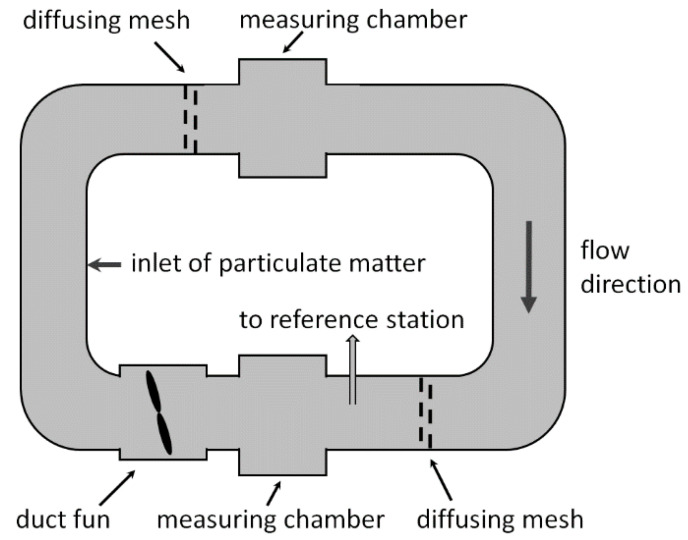
A simplified diagram of the tunnel.

Inside the space tunnel (Figure 2), there are two measuring chambers where tested sensors can be placed. Immediately in front of each measurement chamber, there are diffusing meshes to increase turbulence and mixing of particulate matter in the air.

In this system, the entire spectrum of particles is transferred from the injector into the calibration tunnel, with no filtration or selection of particles of particular sizes. In the process of calibration, some of the particles are deposited on the wall of the tunnel. Therefore, they should be removed after completion of calibration procedure by applying appropriate filters installed in the position of sensors under calibration and ventilating the tunnel with the maximum air flow (in this system: ~7 m·s^−1^) for about 15 min, to clean the pipe up. Shortening of the procedure of pipe clean-up will result in an elevation of the baseline.

**Figure 3:**
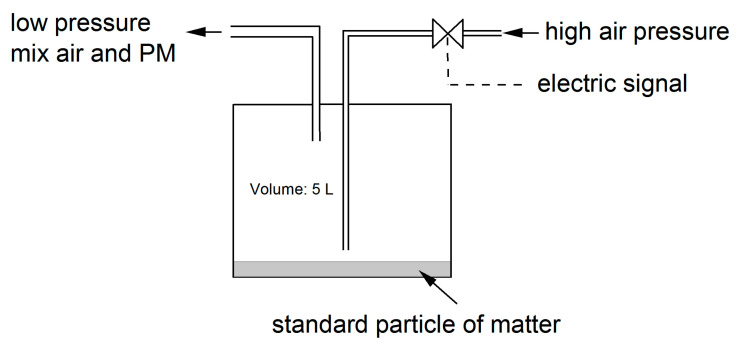
Simplified diagram of the particle matter injector.

The particulate matter injector was built from: (a) a tank (volume 5 L) with particles of matter placed at the bottom of the tank (particle size distribution: 15%@1 µm, 95%@10 µm); (b) an inlet tube with a solenoid valve (TD-06, TEKMA); (c) an outlet tube; (d) and a high-pressure air tank (air compressor or high air pressure installation, ~300 kPa). During every short electric impulse, the solenoid valve is opened for a short period Δt = 2 ms (if Δt < 1.5 ms the valve does not respond), which causes a small amount of the air to enter into the container briefly at high speed, where a cloud of particulate matter was formed. As a result, the pressure slightly increases in the container and the air with particulate matter slowly moves through the short outlet tube (~0.20 m). In this way particulate matter is delivered into the volume of calibrator. A simplified diagram of the particle matter injector is shown in Figure 3.

**Figure 4:**
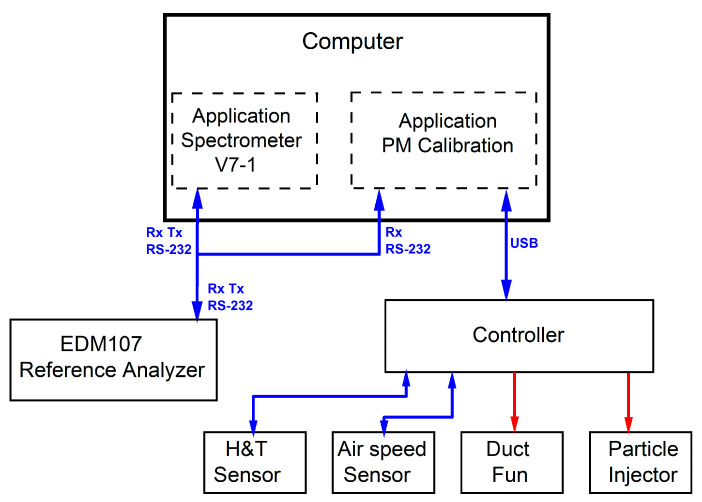
Schematic diagram of information flow in the calibration system. The continuous lines of the rectangle illustrate the hardware and the dashed lines of the rectangle represent software applications on the computer.

A controller built according to a dedicated proprietary idea is the element that enables simultaneous control of devices and reading of data from sensors through one USB interface. The controller consists of the following parts and electronic components: a power supply unit (RS-25-24, MEAN WELL), an analog-to-digital converters (MCP3204-CI/SL, MICROCHIP TECHNOLOGY), a USB-UART converter (FT232, FTDI), an LCD display (DEM16481FGH-PW, DISPLAY ELEKTRONIK), an AVR microcontroller (ATMEGA16, Microchip Technology (Atmel)). The schematic diagram of the functionality of the whole calibration system and the controller is shown in Figure 4.

The calibrator system can perform fully automatic multi-point calibration. The calibration process is carried out by an application called “PM-Calibration” (developed in Python). The input information is a set of data prepared as a dictionary in simple JSON format (datatypes dictionary), which includes information about the number of calibration thresholds, their duration and the value of concentration levels to be calibrated. During the process of calibration, the PM-Calibration program analyzes on-line data obtained from the reference analyzer EDM107 that constantly works with a custom application called “Spectrometer V7-1” provided by the producer (see Figure 4). Obtained data allow PM-Calibration to constantly control the PM injector and provide feedback. The solenoid valve of the particulate matter is opened for short periods of time with a frequency ensuring that the set particulate matter concentration in the calibrator space is maintained.

In general, the system can work properly within the range of PM_1_–PM_10_. The only limitation can be induced by the capacity of the reference station. The EDM 107 station we used allowed us to measure PM_1_, PM_2.5_ and PM_10_, but other commercially available stations can also measure, for instance, PM_5_.

## 3. Results and Discussion

**Figure 5:**
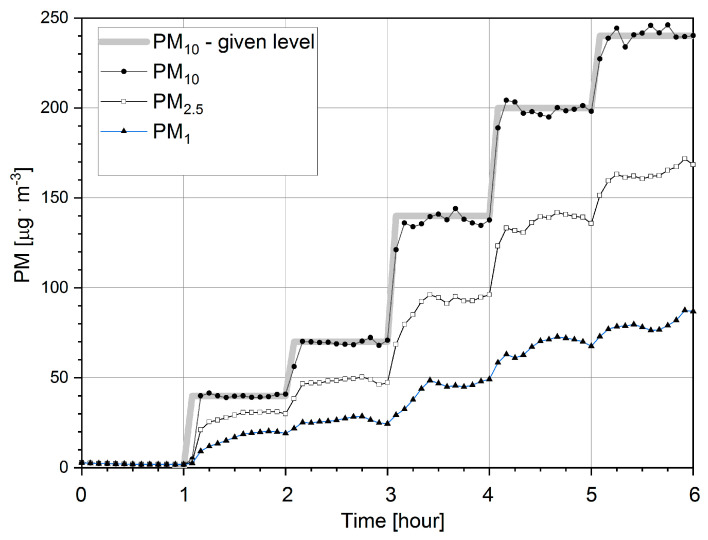
The concentrations of particulate matter PM_1_, PM_2.5_ and PM_10_ recorded as a function of time by reference station EDM 107 during the background level measurement (the first hour) and the programed five levels at 40, 70, 140, 200 and 240 µg · m^−3^. The data presented in this figure are the mean values of 5 min long measurements.

Figure 5 shows the results obtained with the calibrator system as a function of time. The background level is shown at the beginning of the graph (during the first hour), followed by five levels (40, 70, 140, 200 and 240 µg·m^−3^) of particulate matter (PM_10_) concentration which were programmed. The mean deviation from the five applied threshold values amounted to 5.3 µg·m^−^^3^, 2.0 µg·m^−^^3^, 2.8 µg·m^−^^3^, 2.9 µg·m^−^^3^, 3.8 µg·m^−^^3^, respectively. Every PM_10_ level was maintained for one hour. The best stepwise shape of the charts was obtained for index PM_10_, which were used as feedback variable during measurement and control of the process. The presented curves of PM_1_ and PM_2.5_ do not follow precisely the stepwise shape of the charts, as presented in the case of PM_10_ (see Figure 5), since they are not incorporated into the mechanism of regulation with the feedback involving only PM_10_ measurements. The principal distinction lies in the sub- and microscopic behavior of different-sized particles caused by physical and chemical forces [27].

**Figure 6:**
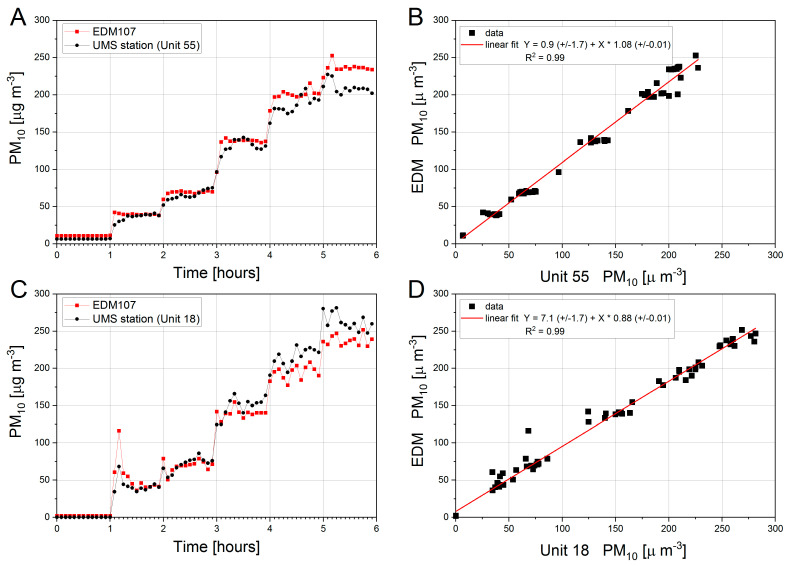
Panels (**A**–**D**). Results of the PM_10_ calibration of two UMS stations (Unit 55, Unit 18) in concentration range from 40 to 240 µg·m^−3^. Panels A and C present PM_10_ values recorded as a function of time by EDM 107 are marked in red and those recorded by UMS stations—in black. Panels B and D present the relation of PM_10_ concentrations obtained from UMS (Unit 55 and Unit 18, respectively) and reference station EDM107. The linear fit was drawn as red line. The data presented in this figures are the mean values of 5 min long measurements.

Figure 6 presents two independent examples of multi-point calibration, where PM_10_ values registered by the reference analyzer (EDM 107) and calibrated UMS were drawn as 5-min average values. The UMS station is permanently installed inside the measurement chamber. Therefore, it is present there during the entire calibration procedure. The sensors are mounted on a metal grille installed inside the measuring chamber. The maximum L × W × H of the sensor in this set-up is: 20 cm × 15 cm × 7.3 cm, respectively. Calibration of PM_10_ index was carried out with five thresholds of particulate matter concentrations: 40, 75, 150, 200 and 240 µg·m^−3^, where—on average—relative humidity (RH) equals to 43% and air temperature 22 °C. All the data obtained in the calibration process were included into curve fitting during calibration. At the beginning of calibration, the air inside calibration tube was clean, both EDM107 and UMS recorded PM_10_ values close to zero. The observed small values of PM_10_ at the beginning of the calibration process result from the presence of dust residues inside the tunnel, which remained after the previous calibration.

In the present paper, we present the outcome of the calibration procedures based on the lowest possible air flow (0.65 m·s^−1^), at which the system remains stable. This air flow is close to air flows occurring in closed systems. That’s why our calibration results could be compared with the results of calibrations obtained by implementing the turbulent air flow method, frequently used by others [21,22]. Our experimental experience with this system allows us to conclude that an increase of air flow reaching up 2 m·s^−1^ does not result yet in development of the cyclonic separation effect, which obviously could affect the calibration outcome. In the future, one should determine the effect of higher air flows up to the current limit of this system (7 m·s^−1^)—see Material and Methods section—on the magnitude of the cyclonic separation effect, in order to optimize this unit for successful sensor calibration at high air flows.

The fan was started up first and, after its start-up, the calibration procedure (t = 0) set the air velocity to 0.65 m·s^−1^. It was followed by the first injection of particulate matter into the calibrator space. The reference analyzer and the calibrated UMS station usually recorded a clear increase in PM_10_ concentration, well above the expected first threshold value 40 µg·m^−3^. The reason for this is the determination of correct coefficients in the feedback mechanism. So, the initial period of particulate matter dosing into the calibration system (about 10 min of the calibration process) was used to establish dynamic equilibrium conditions, feedback parameters in the software and the correct operation of the entire hardware and software feedback.

In the presented examples of calibration (Figure 6, Panels A and C), the control system was programmed to change the threshold PM_10_ concentration level every hour. Thus, the presented 6 h of calibration consists of: (a) 1 h, when the PM_10_ value is at the background level (no injection of particulate, fan running); (b) five 1-h periods with planned particulate matter concentration levels, used to calibrate two UMS stations (Unit 55, Unit 18). Each station was calibrated in a separate measurement procedure. Linear regression is the most common method for calibrating low-cost PM sensors [14,18,19]. The sensors’ outputs are plotted against the outputs from reference instruments, and a fitted equation is used to optimize the accuracy of the sensors’ outputs. In the present study we used also the linear fit. The relationship between concentrations registered by the EDM107 reference analyzer and both calibrated stations is shown in Figure 6 (Panels B and D). Using a model of linear relationship between concentrations, the following were obtained (a) for Unit 55: slope +1.08 (±0.01), intercept +0.9 (±1.7); (b) for Unit 18: slope +0.88 (±0.01), intercept +7.1 (±1.7); all at the level of significance *p* < 0.01. This result agrees with the results presented in details in the previous study [4,28].

Within the entire tested concentration range—from several to 240 µg·m^−3^—it was found that after using linear calibration, the mean error of the calibrated stations was equal to ±9.0 µg·m^−3^ and ±9.3 µg·m^−3^, for Unit 55 and Unit 18, respectively. In contrast, at the beginning the error reached values ±19.2 µg·m^−3^ and ±28.8 µg·m^−3^ in the upper threshold of the tested range for Unit 55 and Unit 18 respectively. In station 55 the sum of the absolute residuals was found for PM concentrations corresponding to the first threshold (40 µg·m^−3^) and maximal sum of the absolute residuals was found for PM concentrations corresponding to the last threshold (240 µg·m^−3^), where—as for station 18—the minimal sum of the absolute residuals was present at the third PM threshold (140 µg·m^−3^) and the maximal sum of absolute residuals was found at the first PM threshold (40 µg·m^−3^). As presented in Figure 6, Panels A and C, the time responses of the UMS and the EDM are very similar. Moreover, the EDM reproducibility (Figure 5 and Figure 6) is very similar as well.

It is worth mentioning that the presented calibration system is successfully used to maintain our continuous air quality measurements within the framework of the scientific project known as the Storm&DustNet implemented at the Jagiellonian University in Kraków (Poland), as described previously [4].

## 4. Conclusions

This study presents a simple and low-cost system dedicated for calibration of low-cost suspended particulate matter sensors, with programmed air velocity and known air temperature and humidity. It allows one to calibrate PM sensors regardless of current field conditions using standard solid particles (i.e., standard quartz dust). It should be noted that this is essential for a system composed of a large number of low-cost sensors.

In our opinion, the presented new calibration system opens the possibility of enhancing the quality of measurements with low-cost PM sensors. This could be achieved by performing regular calibration procedures that allow the functional state of sensors to be diagnosed.

In addition, this study examined accuracy of two low-cost UMS stations measuring PM_10_ within concentration range of up to 240 µg·m^–3^. The presented final results of calibration of both stations (see Figure 6) show that the calibrated readings of both stations are characterized by similar accuracy of about ±9.0 µg·m^–3^ within the entire range of PM_10_ concentrations under analysis.
